# Readability of Arabic Web-Based Information on Nitrous Oxide Sedation for Pediatric Dental Patients: A Cross-Sectional Analysis

**DOI:** 10.7759/cureus.109084

**Published:** 2026-05-18

**Authors:** Rahaf Alolayan

**Affiliations:** 1 Pediatric Dentistry, Private Practice, Qassim, SAU

**Keywords:** health literacy, inhalation sedation, nitrous oxide, online health information, parental understanding, pediatric dentistry, readability

## Abstract

Background: Nitrous oxide inhalation sedation is a cornerstone of behavior management in pediatric dentistry, widely used to reduce anxiety and enhance cooperation in child dental patients. Online health information significantly influences parental understanding and decision-making regarding their children's dental treatment. However, the readability of Arabic web-based information on this topic remains unexplored.

Objective: To assess the readability of Arabic web-based information on nitrous oxide sedation in pediatric dentistry using the Gunning Fog Index (GFI), with implications for parental understanding.

Methods: A cross-sectional study was conducted in May 2026. The first 100 search results were screened from Google, Yahoo, and Bing using four Arabic search phrases. Eligible Arabic-language websites were analyzed using the online Gunning Fog Index calculator. Descriptive statistics were calculated, and readability was stratified by search engine and content source. The exact Arabic search terms used were "أكسيد النيتروز للأطفال" (nitrous oxide for children), "أكسيد النيتروز" (nitrous oxide), "الغاز الضاحك" (laughing gas), and "التخدير باستنشاق أكسيد النيتروز" (nitrous oxide inhalation sedation). The searches were conducted in May 2026 using standard browser settings (Google Chrome in incognito mode; Google LLC, Mountain View, CA, USA)) to minimize personalization effects.

Results: Thirty-two websites met the inclusion criteria. The mean GFI was 9.92 ± 4.42 (range: 2.00-19.00), corresponding to a high school sophomore reading level. Only 12.5% (4/32) of websites met the American Medical Association's (AMA) recommended reading level (GFI ≤6). Most content originated from private dental practices (65.6%, 21/32). Public journals demonstrated significantly higher GFI scores (mean: 16.00 ± 3.46) compared to private practices (9.32 ± 3.96).

Conclusion: Arabic web-based information on nitrous oxide sedation for pediatric dental patients exceeds recommended readability levels for parental education materials. Pediatric dentists and content creators should simplify online health information to improve parental comprehension.

## Introduction

Nitrous oxide inhalation sedation, commonly known as "laughing gas," is a safe and effective anxiolytic technique widely employed in dental and medical settings, particularly for pediatric patients and those with dental anxiety [1,2]. Its rapid onset, ease of administration, and favorable safety profile have made it a cornerstone of conscious sedation in ambulatory care settings [3]. 

In the digital age, patients and caregivers increasingly rely on online resources to gather health information before medical and dental procedures [4,5]. Studies indicate that 72% of internet users search for health-related information online, and this trend is particularly pronounced among parents seeking information about pediatric procedures [6]. The quality and readability of this information directly impact patient understanding, informed consent, and treatment acceptance [7].

Health literacy, the ability to obtain, process, and understand basic health information, is a critical determinant of health outcomes [8]. The American Medical Association (AMA) and the National Institutes of Health (NIH) recommend that patient education materials be written at or below the sixth-grade reading level to ensure accessibility across diverse literacy levels [9,10]. However, numerous studies have demonstrated that online health information frequently exceeds these recommendations, potentially limiting comprehension among individuals with lower health literacy [11-13].

While several studies have evaluated the readability of English-language dental and medical content online [14-16], limited research exists on Arabic-language health information. The Arabic-speaking population exceeds 420 million individuals globally, representing diverse geographic, cultural, and educational backgrounds [17]. Assessing the readability of Arabic health content is essential to ensure equitable access to comprehensible health information.

To our knowledge, no previous study has examined the readability of Arabic web-based information on nitrous oxide inhalation sedation. This study aims to evaluate the readability of such content using the Gunning Fog Index (GFI) [18] and to compare readability across different search engines and content sources.

The objective of this study was to evaluate the readability of Arabic web-based information on nitrous oxide sedation for pediatric dental patients using the GFI and to compare readability levels against the AMA-recommended sixth-grade reading level in order to assess the accessibility of online health information for Arabic-speaking parents and caregivers.

## Materials and methods

Study design

This cross-sectional study was conducted in May 2026 to assess the readability of Arabic web-based information on nitrous oxide inhalation sedation. The study methodology was designed to simulate the typical information-seeking behavior of Arabic-speaking patients and caregivers.

Search strategy

Three major search engines, Google, Yahoo, and Bing, were queried using four Arabic search phrases commonly used by patients: Four Arabic search phrases were used: "أكسيد النيتروز للأطفال" (nitrous oxide for children), "أكسيد النيتروز" (nitrous oxide), "الغاز الضاحك" (laughing gas), and "التخدير باستنشاق أكسيد النيتروز" (nitrous oxide inhalation sedation). The searches were conducted in May 2026 using Google Chrome in incognito mode (Google LLC, Mountain View, CA, USA) to minimize personalization effects.

The first 100 search results from each search engine were systematically screened. Searches were performed using standard browser settings without personalization to ensure reproducibility.

Website screening was performed by a single reviewer following predetermined criteria. A random sample of 10% of excluded websites was reviewed by a second independent reviewer to ensure consistency of application of inclusion/exclusion criteria.

Inclusion and exclusion criteria

Websites were included if they: (1) contained Arabic-language content, (2) provided information about nitrous oxide inhalation sedation, and (3) were publicly accessible without registration or payment. Websites were excluded if they: (1) were duplicates, (2) contained primarily non-text content (videos, podcasts), (3) were broken links or inaccessible, (4) required authentication, or (5) were commercial product advertisements without educational content.

Readability assessment

Text extraction procedure: The main body content of each website was copied, excluding navigation menus, advertisements, headers, and footers. Only Arabic text directly related to nitrous oxide sedation was included. The extracted text was then input into the GFI online calculator for automated analysis [19].

Text extraction was performed by copying the main body content of each website, excluding navigation menus, advertisements, headers, and footers. Only Arabic text related to nitrous oxide sedation information was included in the readability analysis. The extracted text was then assessed using the GFI through the online calculator for automated readability scoring [19].

The GFI was selected as the readability metric due to its widespread use in healthcare literature and validated application across multiple languages [18,20]. The GFI estimates the years of formal education required to comprehend a text on first reading. It is calculated using the formula:

$\mathrm{GFI} = 0.4 \times \left[\left(\frac{\mathrm{words}}{\mathrm{sentences}}\right) + 100 \times \left(\frac{\mathrm{complex\ words}}{\mathrm{words}}\right)\right]$

Complex words are defined as those containing three or more syllables. Text content from each website was extracted and analyzed using the online GFI calculator [19], which has been validated for Arabic text analysis.

Data collection

For each included website, the following data were extracted: (1) website URL, (2) search engine source, (3) GFI score, (4) year of publication or last update, and (5) content source category (private practice, health informatics website, public journal, public magazine, or encyclopedia). Websites were categorized based on their primary organizational affiliation and purpose.

Statistical analysis

Descriptive statistics were calculated for all variables. Continuous data (GFI scores) were expressed as mean ± standard deviation (SD), median, range, and interquartile range (IQR). Categorical data were presented as frequencies and percentages. A one-sample t-test was performed to compare the mean GFI score against the AMA-recommended level of 6.0. A chi-square (χ²) goodness-of-fit test was used to assess whether the proportion of websites meeting AMA recommendations differed from expected values. Statistical significance was set at p < 0.05. All analyses were conducted using IBM SPSS Statistics software (version 26, IBM Corp., Armonk, NY, USA).

## Results

Website characteristics

Of the 100 screened search results, 32 websites met the inclusion criteria and were included in the analysis. The majority of websites were retrieved from Google (n=21, 65.6%), followed by Yahoo (n=7, 21.9%) and Bing (n=4, 12.5%). Table 1 summarizes the distribution of websites by search engine and content source.

**Table 1:** Distribution of Websites by Search Engine and Content Source Data are presented as N (%). Chi-square test was used to assess the distribution. Statistical significance is set at p < 0.05.

Category	n	Percentage (%)
Search Engine		
Google	21	65.6
Yahoo	7	21.9
Bing	4	12.5
Content Source		
Private practice	21	65.6
Health informatics website	4	12.5
Public journal	3	9.4
Public magazine	2	6.3
Other	2	6.3

The majority of content originated from private dental and medical practices (n=21, 65.6%), followed by health informatics websites (n=4, 12.5%), public journals (n=4, 10.5%), public magazines (n=2, 6.3%), and encyclopedias (n=1, 3.1%). χ² goodness-of-fit testing revealed that the distribution of source types was significantly non-uniform (χ² = 56.50, df = 4, p < 0.001), indicating dominance of commercial dental practice websites in the available Arabic online content about nitrous oxide sedation. Publication years ranged from 2017 to 2026, with the highest proportion of websites published in 2025 (n=13, 40.6%).

Overall readability analysis

The overall mean GFI score was 9.92 ± 4.42, with a median of 8.62 (range: 2.00-19.00). The IQR was 5.13 (Q1: 6.87, Q3: 12.00). These values correspond to approximately a high school sophomore reading level, substantially exceeding the AMA-recommended sixth-grade level. Table 2 presents comprehensive descriptive statistics for the overall sample.

**Table 2:** Descriptive Statistics of Gunning Fog Index (GFI) Scores (N=32) Data presented as mean ± SD, median (IQR), and range. One-sample t-test (t = 5.02, df = 31) was used to compare the mean GFI against the AMA recommendation (6.0). Statistical significance set at p < 0.001

Statistic	Value
Mean ± SD	9.92 ± 4.42
Median	8.62
Range	2.00 – 19.00
Interquartile range (IQR)	5.13
Websites at American Medical Association (AMA)-recommended level (GFI ≤6), n (%)	4 (12.5%)
Websites exceeding high school level (GFI >12), n (%)	6 (18.8%)

Only n = 4 (12.5%) achieved the AMA-recommended readability level (GFI ≤ 6), while n = 6 (18.8%) exceeded a high school reading level (GFI > 12), indicating college-level or postgraduate complexity. Statistical testing revealed that the mean GFI score was significantly higher than the AMA-recommended level of 6.0 (t = 5.02, p < 0.001, 95% CI: 8.39-11.45). χ² goodness-of-fit testing demonstrated that significantly fewer websites met AMA readability recommendations than would be expected (χ² = 18.00, df = 1, p < 0.001).

Readability category distribution

Websites were stratified into five readability categories based on GFI scores (Table 3). The distribution revealed that most websites (68.8%) fell within middle school to high school reading levels. Notably, 15.6% of websites required postgraduate-level reading ability (GFI > 16), representing a substantial barrier to comprehension for the general population.

**Table 3:** Distribution of Websites by Readability Category Data presented as N (%). Chi-square goodness-of-fit test (χ² = 18.00, df = 1) was used to assess proportion meeting AMA standards. Statistical significance set at p < 0.001.

Readability Category (Gunning Fog Index Range)	n	Percentage (%)
Easy/American Medical Association (AMA)-Recommended (≤6)	4	12.5
Middle School (7–8)	11	34.4
High School (9–12)	11	34.4
College (13–16)	1	3.1
Postgraduate/Very Difficult (>16)	5	15.6

## Discussion

This study represents a comprehensive assessment of the readability of Arabic web-based information on nitrous oxide inhalation sedation. Our findings reveal that the overwhelming majority of online content exceeds recommended readability levels for patient education materials, potentially limiting accessibility for individuals with lower health literacy.

The mean GFI score of 9.92 indicates that Arabic web content on nitrous oxide sedation requires approximately a high school sophomore reading level, significantly higher than the AMA-recommended sixth-grade level [9]. Only 12.5% of websites met this benchmark, suggesting a substantial mismatch between content complexity and target audience literacy levels. This finding aligns with previous studies evaluating English-language dental health information, which similarly reported readability levels exceeding recommendations [21,22]. 

The high variability in readability scores (range: 2.00-19.00, SD: 4.42) suggests inconsistent content quality across sources. The majority of content originated from private dental and medical practices (65.6%), which showed moderate-to-high complexity. This finding is particularly significant given that patients frequently consult practice websites for procedural information. The moderate-to-high complexity of this content may contribute to patient anxiety, misunderstandings, and suboptimal informed consent [23]. Healthcare providers should prioritize simplifying online content to enhance patient comprehension and engagement.

The implications of these findings are substantial. Health literacy is a critical determinant of health outcomes, influencing treatment adherence, disease management, and healthcare utilization [24,25]. When online health information exceeds patients' comprehension levels, it can exacerbate health disparities, particularly among vulnerable populations with limited education [26]. In the context of nitrous oxide sedation, a procedure often performed on children, caregivers' ability to understand procedural risks, benefits, and alternatives is essential for informed decision-making.

Study limitations

Several limitations should be acknowledged. First, the GFI, while widely used, does not account for cultural context, domain-specific terminology familiarity, or reader motivation, all of which influence comprehension. Second, our sample was limited to the first 100 search results, potentially excluding relevant content ranked lower. Third, readability scores provide only one dimension of content quality; accuracy, completeness, and cultural appropriateness are equally important but were not assessed in this study. Finally, this study focused exclusively on Arabic-language content; comparisons with other languages were beyond our scope. Additionally, readability scores provide an estimate of text difficulty but may not fully reflect actual reader comprehension, particularly for Arabic speakers with varying literacy levels and health literacy backgrounds.

It is important to note that while readability scores indicate text complexity, this study did not directly measure actual patient or caregiver comprehension. Future research should include direct assessment of reader understanding to validate these readability-based findings and their clinical implications.

## Conclusions

Arabic web-based information on nitrous oxide inhalation sedation generally exceeds recommended readability levels for patient education, with a GFI far above the AMA-recommended sixth-grade level. Only some of the websites met accessibility standards, highlighting a critical gap between available content and patient literacy needs. Content from public journals demonstrated the highest complexity, while public magazines approached recommended levels, suggesting that simplified health communication is achievable in Arabic.

Healthcare providers, content creators, and digital health platforms must prioritize plain language, simplified sentence structures, and patient-centered design to improve accessibility. Enhanced readability of online health information is essential to empower patients and caregivers with the knowledge necessary for informed decision-making, ultimately reducing health disparities and improving outcomes in Arabic-speaking populations.
